# Performance Evaluation of a Magnetically Actuated Capsule Microrobotic System for Medical Applications

**DOI:** 10.3390/mi9120641

**Published:** 2018-12-04

**Authors:** Qiang Fu, Songyuan Zhang, Shuxiang Guo, Jian Guo

**Affiliations:** 1 Tianjin Key Laboratory for Control Theory & Application in Complicated Systems and Biomedical Robot Laboratory, School of Electrical and Electronic Engineering, Tianjin University of Technology, Binshui Xidao 391, Tianjin 300384, China; gj15102231710@163.com; 2 State Key Laboratory of Robotics and System, Harbin Institute of Technology, Harbin 150001, China; 3 School of Life Science, Key Laboratory of Convergence Medical Engineering System and Healthcare Technology, The Ministry of Industry and Information Technology, The Institute of Advanced Biomedical Engineering System, Beijing Institute of Technology, Beijing 100081, China; guoshuxiang@hotmail.com; 4 Faculty of Engineering, Kagawa University, Takamatsu 761-0396, Japan

**Keywords:** magnetic actuated capsule microrobotic system, magnetically actuated hybrid microrobot, rotational magnetic field plane, magnetic field changing frequency

## Abstract

The paper aims to propose a magnetic actuated capsule microrobotic system, which is composed of a magnetically actuated microrobot with a screw jet mechanism, a driving system, and a positioning system. The magnetically actuated microrobot embedded an O-ring magnet as an actuator has potential for achieving a particular task, such as medical diagnose or drug delivery. The driving system composes of a three axes Helmholtz coils to generate a rotational magnetic field for controlling the magnetically actuated microrobot to realize the basic motion in pipe, e.g., forward/backward motion and upward/downward motion. The positioning system is used to detect the pose of the magnetically actuated microrobot in pipe. We will discuss the shape of the Helmholtz coils and the magnetic field around the O-ring magnet to obtain an optimal performance of the magnetically actuated microrobot. The experimental result indicated that the microrobot with screw jet motion has a flexible movement in pipe by adjusting the rotational magnetic field plane and the magnetic field changing frequency.

## 1. Introduction

Wireless capsule endoscope has been widely used to achieve a medical procedure in clinical applications, such as medical diagnoses, treatments, and noninvasive therapy [1,2,3,4,5]. Compared with the tedious and cumbersome insertion traditional endoscope, it has great potential to safe, reliable, painless techniques to achieve the task in a complex environment [6,7,8]. Given Imaging of Israel [9] proposed a wireless capsule endoscopy (M2A capsule endoscopy), which move smoothly and painlessly in the gastrointestinal (GI) tract by the natural peristalsis of the gastrointestinal tract. However, it still has limitations, e.g., flexible motion, due to the passive movement of the capsule endoscope. To solve these problems, many control methods were proposed for realizing the real-time control or active movement of the capsule endoscope, e.g., shape memory alloy (SMA) actuator and motors [10,11,12,13].

With the rapid progress of microrobot technologies, the microrobot manipulated by the external magnetic field becomes more popular in medical applications. It has the characteristics of flexibility and good response. Furthermore, different kinds of control system were proposed to generate the magnetic field to control the microrobot, such as, gradient magnetic field, uniform magnetic field and oscillation magnetic field [14,15,16,17,18,19]. To manipulate the magnetic microrobot, Choi et al. developed an electromagnetic actuation (EMA) system to generate gradient and uniform magnetic field by saddle coils [20]. Guo et al. proposed a kind of microrobot fish-like microrobot which was driven by an oscillating magnetic field generated by the solenoid coils [21]. And they controlled the microrobot to realize the flexible motion by a MTx sensor, which mimics the natural lashing of the fish. However, the movement path of microrobot is limited because of the shape of the solenoid coils. Qan et al. proposed a control system composed of an electromagnet to control the magnetically actuated microrobot [22,23,24]. The movement path of microrobot is not limited. But the movement of the microrobot is unstable due to asymmetric of the electromagnet. Fountain et al. proposed a magnetic helical microrobot. It is manipulated by a single rotating permanent-magnet, which generates a non-uniform magnetic field [25]. Sitti et al. proposed a magnetically actuated soft capsule endoscope (MASCE) as a miniature mobile robot platform for diagnostic in medical applications [26]. Okada et al. also proposed a magnetic microrobot with screw motion inspired by drill [27]. It realized flexible motion in pipe. But this kind of microrobot maybe brings damage to intestinal surface due to the exposed screw structure. Steager et al. proposed an electromagnetic actuation (EMA) system composed four electromagnetic coils to manipulate a microrobot [28]. However, the EMA system can control the microrobot on two-dimensional planes. According to the previous research, we proposed a magnetically actuated capsule microrobotic system which has only three stationary pairs of Helmholtz coils (6-Helmholtz coil). Through the control of the current value applied to each coil in our proposed microrobotic system, a magnetic capsule microrobot with screw jet motion can be manipulated to a desire direction on the horizontal plane and vertical plane.

This paper is structured as the following. Firstly, we introduce the configuration of the magnetic actuated capsule microrobotic system in Section 2. Secondly, we propose a screw jet type microrobot and discussed the screw jet mechanism in Section 3. In Section 4, we carry out the evaluation experiments of the microrobot using our proposed microrobotic system and analyze the experimental results. Finally, conclusions and future work are illustrated.

## 2. Conceptual Design of Magnetic Actuated Capsule Microrobotic System

### 2.1. Magnetic Actuated Capsule Microrobotic System

**Figure 1:**
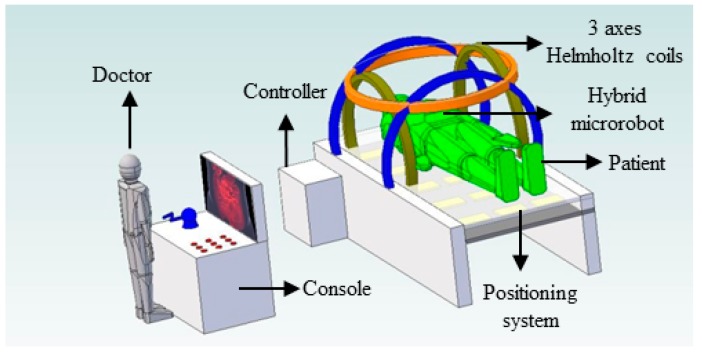
Overall of the control system.

**Figure 2:**
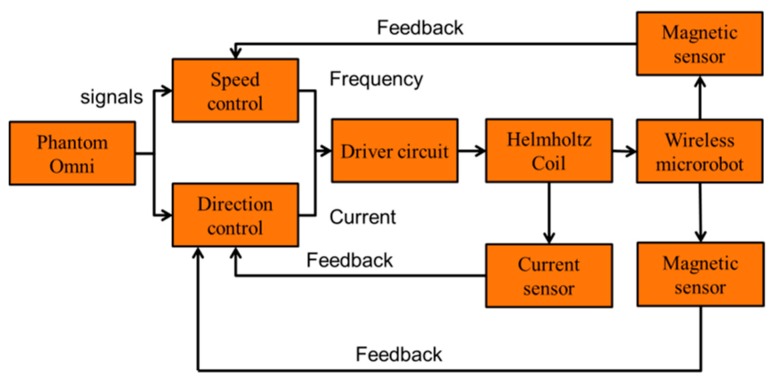
Algorithm design of the magnetically actuated capsule microrobotic system.

The magnetic actuated capsule microrobotic system provides telepresence by allowing a doctor to remotely control a magnetic actuated capsule microrobot through a master device. This causes less pain to the patients and there will be less tissue trauma, thus reducing hospitalization time and enhancing recovery. The conceptual diagram illustrates a method of examining a tubular digestive system, as shown in Figure 1. The algorithm design of the magnetic actuated capsule microrobotic system is shown in Figure 2. On the master side, the doctor views a monitor which is produced by a CT-scan and operates the wireless microrobot to detect or treat the disease with an unknown and dynamic environment. The control instructions are transmitted to the slave side. On receiving instructions, the slave mechanisms control the wireless capsule microrobot. The monitor can also show the data calculated from the position system for obtaining the real-time position and posture of the robot. The positioning system detects the magnetic field intensity generated by the O-ring magnet, which is installed inside the magnetically actuated capsule microrobot. We chose 6 parameters, which are *x*, *y*, *z*, roll, pitch and yaw as output of the position parameter, to calculate the position of the microrobot. We used a least square method to solve the inverse problem. The positioning system helps us to realize the close-loop control and ensure the robot achieves the task. Consequently, the doctor appears to accurately control the position and posture of the wireless microrobot in human body.

### 2.2. Magnetically Actuated Capsule Microrobot

**Figure 3:**
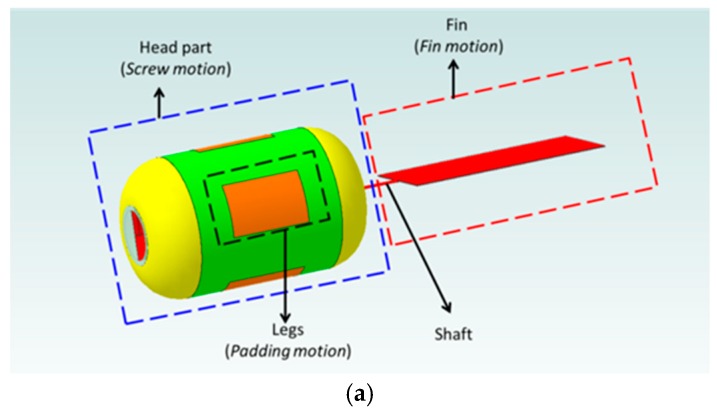

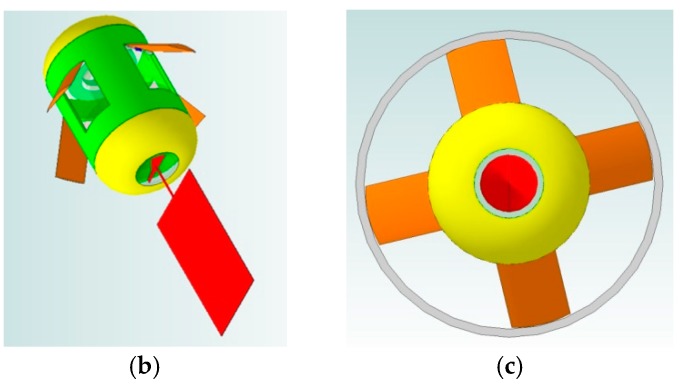
(**a**) Magnetically actuated capsule microrobot with leg close, (**b**) Magnetically actuated capsule microrobot with leg open, (**c**) Stop motion by the open leg in pipe.

Up to now, various kinds of microrobot have been developed by our group [29,30,31]. Especially, the microrobot driven by a magnetic field has a potential in the biomedical applications. Based on the research results, a magnetically actuated capsule microrobot with hybrid motion has been proposed. It has a compact structure with a wireless power supply, characteristics of multi-functions, controllability, and stability. The magnetically actuated capsule microrobot is composed of two driven mechanical structures, screw head (screw structure) which generates the screw jet motion, fin which generates the fin motion, as shown in Figure 3a, and a stop mechanism which can stop at a point through opening the leg of the magnetically actuated capsule microrobot in the pipe as shown in Figure 3b,c. In order to obtain the stable motion, we specially designed four legs on the microrobot surface.

**Figure 4:**
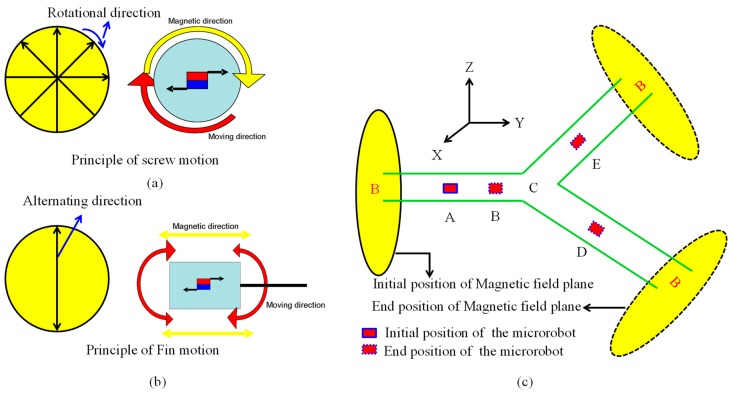
Movement principle of the magnetically actuated capsule microrobot with hybrid motion.

The Figure 4 shows the movement principle of the magnetically actuated capsule microrobot with hybrid motions. While the magnetically actuated microrobot is placed inside of the rotational external magnetic field, the magnetically actuated microrobot with magnet materials embedded (e.g., permanent magnet, magnet sheet) can rotate synchronously with the changing frequency of the external magnetic field, because the pure magnetic moment is generated as a dipole of magnet attempts to align with the local magnetic field. The screw structure generates the propulsive force due to pushing back the fluid, as shown in Figure 4a. When an alternating magnetic field parallel to the moving direction is applied, an impelling force to a permanent magnet rotates and vibrates the connected fin, as shown in Figure 4b. We assume the microrobot move from an initial position (Point A) to end position (Point D) as shown in Figure 4c, it should be moved following these steps:

Step 1: While the microrobot with screw motion moves forwardly in the Y-direction, we control the external magnetic field by clockwise rotating in the X-Z plane. Otherwise, we control the external magnetic field by counter-clockwise rotating in the X-Z plane, the hybrid microrobot moves backwardly in the Y-direction. If an alternating magnetic field is generated, the microrobot can move to forward by the fin motion.

Step 2: While the microrobot moves to the branch point C, the magnetic field plane is rotated to perpendicular to the locomotion direction of the microrobot, and then the locomotion direction of the microrobot turn to the right.

Step 3: And then, the microrobot move to point D with the screw motion or fin motion, while the rotational magnetic field or alternating magnetic field is generated in the plane of perpendicular to the moving direction.

Following the procedure noted above, by adjusting the direction of the magnetic field in any plane, the microrobot can realize the forward motion and backward motion in the perpendicular to the rotational magnetic field.

### 2.3. Control Method

For medical application of the clinical examination, the coil should have enough inner space to accommodate a human subject and reduce exposed radiation. In general, circular and square Helmholtz coils are used to produce a uniform magnetic field. Therefore, it is important to analyze the performance of the shape of the Helmholtz coil. Based on the Biot-Savart Law, a stationary electric current (*I*) through the polygonal coil, the magnetic field (*B*) at position (*r*) in three-dimensional space is defined by Equations (1) and (2).

(1) $$\vec{B}=\mu_{0}H=\frac{\mu_{0}I}{4\pi }\int_{L}^{}\frac{dl\times {\vec{e}}_{r}}{r^{2}}$$

(2) $$\underset{n\to \infty }{\mathrm{Lim}}B=\frac{\mu_{0}nI}{4\pi r}\times \sin\frac{2\pi }{n}$$

where, *n* is the number of sides of the polygonal Helmholtz coil. *L* is the current flow the path. is the unit vector.

**Figure 5:**
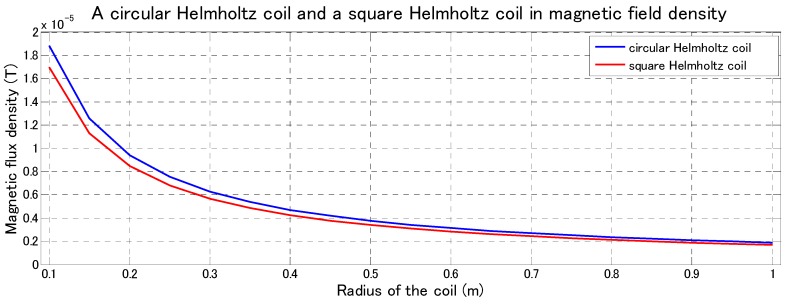
Simulation results of the magnetic flux density.

Furthermore, based on the finite element method (FEM), the magnetic flux density of the circular Helmholtz coils compared with the square Helmholtz coils is simulated by PHOTO-Series software. We designed two types of Helmholtz coils, circular Helmholtz coils and square Helmholtz coils with the same parameters electric current *I*. Here, we assumed the electric current *I* = 1 A and *μ*_0_ = 4π × 10^−^^7^ respectively. The radius of the coils is changed from 0.1 m to 1 m. The magnetic flux density shows the change slowly from 0.4 m to 1 m. The relationship between the radius of the Helmholtz coil and the magnetic flux density is shown in Figure 5. As a result, under conditions of the same radius, produced magnetic flux density of the circular Helmholtz is larger than square Helmholtz coil.

When n tends to infinity, the magnetic flux density in the center of the polygonal Helmholtz coil is maximum value. In other words, magnetic flux density generated by the circular Helmholtz coils is greater than magnetic flux density generated by the polygonal Helmholtz coils with the same R.

**Figure 6:**
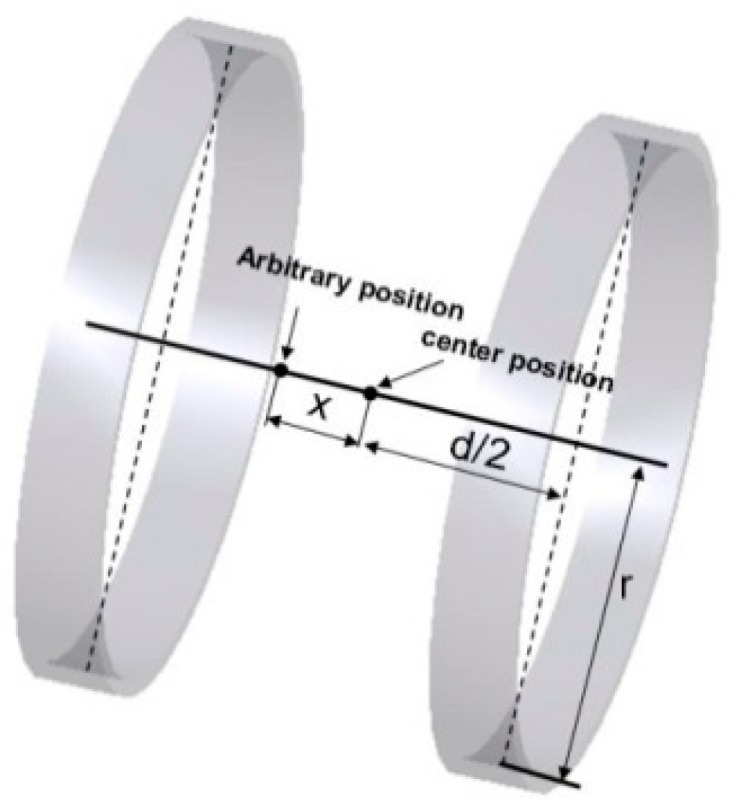
Single Helmholtz coils.

Based on the results of this modeling analysis, it is better to choose circular Helmholtz coil rather than polygonal Helmholtz coils for the external electromagnetic system due to the requirement for higher magnetic field intensity. Therefore, in our research, the circular Helmholtz is used to control the magnetically actuated microrobot. The basic premise of a Helmholtz coil is that it produces a uniform magnetic field in its center plane, as shown in Figure 6. Magnetic flux density is directly proportional to the number of turns in the coils and the current applied to them. The relationship of Helmholtz coils between magnetic flux density and current is given by the Equation (3) [29].

(3) $$B=\frac{IN\mu_{0}R^{2}}{2}\left\{\frac{1}{{\left[R^{2}+{\left(\frac{d}{2}-x\right)}^{2}\right]}^{\frac{3}{2}}}+\frac{1}{{\left[R^{2}+{\left(\frac{d}{2}+x\right)}^{2}\right]}^{\frac{3}{2}}}\right\}$$

where, *B* is the magnetic flux density, at any point on the axis of the Helmholtz coils. *μ*_0_ is 4π × 10^−7^ N/A^2^. *I* is the current of coil in amperes. N is the number of turns of coil. *R* is the radius of the coil. *d* is the distance between pair coils. *x* is the arbitrary position from the center position of the pair coils.

### 2.4. Performance Evaluation

**Figure 7:**
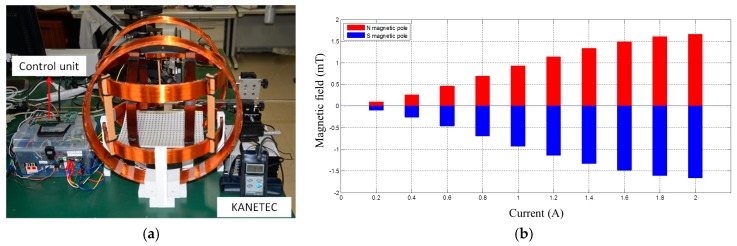
Measurement results (**a**) Measurement system; (**b**) Relationship between the current and magnetic field.

We measured the magnetic field in the region of interest of the Helmholtz coils using our proposed measurement system [32]. The Gauss meter (TM701, KANETEC, Nagano, Japan) was used to measure the magnetic field between each Helmholtz coil, as shown in Figure 7a. The magnetic field can be measured between the range of 0 to 2 A with step 0.2 A. The measurement results are shown in Figure 7b. The experimental results indicated that our design Helmholtz coils can generate a stable magnetic field, while the magnetic field direction is changing. And also, the relationship between the current and magnetic field is the linear, which is very close to our simulated results [29]. It generated the maximum magnetic field in 2 mT with 2 A.

**Figure 8:**
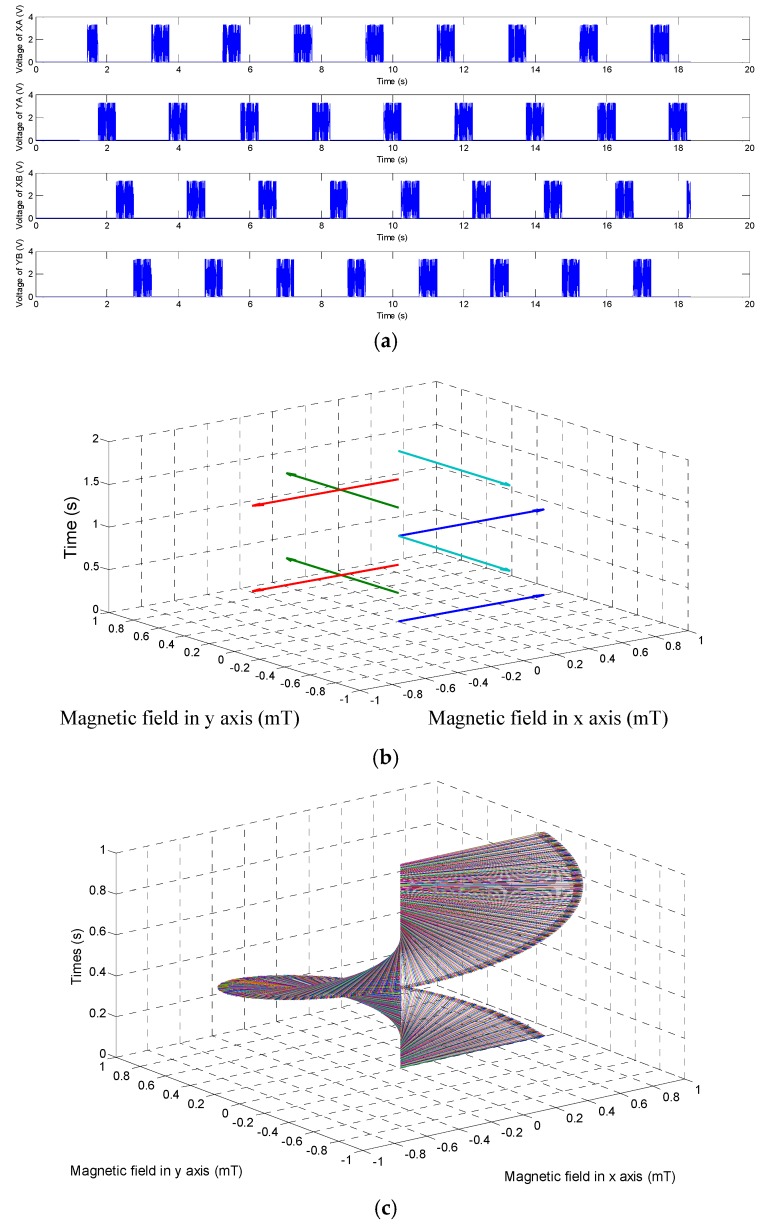
(**a**) Input square wave signals of driving system; (**b**) Rotational magnetic field with square wave signals, (**c**) Rotational magnetic field with sine wave signals.

It is very important that how to generate the rotational magnetic field inside three axes Helmholtz coils. We assumed the microrobot move along the Z direction. Three axes Helmholtz coils should generate a rotational magnetic field in the X-Y plane. While the system outputting the driving signals, as shown in Figure 8a, a rotational magnetic field generates. Through changing the frequency of input square wave signals, we can get a rotational magnetic field and the magnetic changing frequency is adjusted to realize the speed control of the microrobot, as shown in Figure 8b. In order to obtain a stable motion, a sine wave is chosen as input signal for our proposed microrobotic system, as shown in Figure 8c.

## 3. Screw Jet Motion Mechanism

### 3.1. Magnetically Actuated Capsule Microrobot with Screw Jet Motion

**Figure 9:**
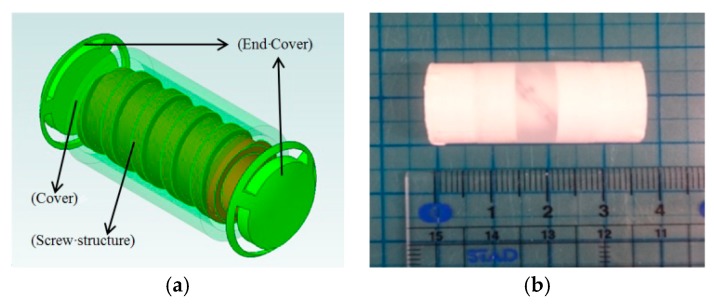
Magnetically actuated capsule microrobot with screw jet motion (**a**) Structure of the microrobot; (**b**) Prototype of the microrobot.

Based on previous researches, we have evaluated the performance of the magnetically actuated capsule microrobot with fin motion and padding motion in different conditions. It realized the basic motion. But it is not enough, because the fin motion only realized the forward motion. The screw jet motion is very important in the medical application. Therefore, we made a microrobot with screw jet motion to evaluate the performance of the microrobot, as shown in Figure 9. The magnetic actuated microrobot is composed of an O-ring magnet as the actuator and a bare propeller fitted with a non-rotating nozzle, which can improve the efficiency of the microrobot with a limited diameter. When the microrobot rotates in the fluid, the propeller pushes the fluid backward while generating a reaction force, therefore the microrobot can move forward. The O-ring magnet is fitted inside the screw structure by a strong adhesive. We designed the body of the microrobot by 3D printer Specification of the microrobot is shown in the Table 1.

A model is built to analyze the magnetic field distribution of O-ring magnet. The magnetic field of the O-ring magnet is calculated by Equations (4) and (5).

(4) $$\oint_{L}B\cdot dl=\mu_{0}\sum I$$

(5) $$dB=\frac{Idl\times r_{0}}{4\pi r^{2}}$$

The magnetic field of the magnet in the center (*B_o_*) is given by Equation (6)

(6) $$B_{o}=\mu_{0}H_{o}=\frac{\mu_{0}I}{2R}$$

The magnetic field of the magnet in the radius direction (*B_R_*) is given by Equation (7).

(7) $$B_{R}=\mu_{0}H_{R}=\frac{\mu_{0}IS}{2\pi {\left(R^{2}+x^{2}\right)}^{3/2}}$$

The magnetic density is any position (*x*, *y*, *z*) is defined by Equations (8) and (9).

(8) $$\left[\begin{array}{c} H_{x}\\ H_{y}\\ H_{z} \end{array}\right]={\left[\begin{array}{ccc} a_{11} & a_{12} & a_{13}\\ a_{21} & a_{22} & a_{23}\\ a_{31} & a_{32} & a_{33} \end{array}\right]}^{-1}\left[\begin{array}{c} H_{X}\\ H_{Y}\\ H_{Z} \end{array}\right]$$

(9) $$\{\begin{array}{l} a_{11}=\cos\alpha \cos\beta \\ a_{12}=-\sin\alpha \cos\gamma +\cos\alpha \sin\beta \sin\gamma \\ a_{13}=\sin\alpha \sin\gamma +\cos\alpha \sin\beta \cos\gamma \\ a_{21}=\sin\alpha \cos\beta \\ a_{22}=\cos\alpha \cos\gamma +\sin\alpha \sin\beta \sin\gamma \\ a_{23}=-\cos\alpha \sin\gamma +\sin\alpha \sin\beta \cos\gamma \\ a_{31}=-\sin\beta \\ a_{32}=\cos\beta \sin\gamma \\ a_{33}=\cos\beta \cos\gamma \end{array}$$

where, *H_X_*, *H_Y_* and *H_Z_* are the unit vector of the magnetic density. *α*, *β*, and *γ* are the angle with the *x*-axis, *y*-axis and *z*-axis.

The double curl equation of static magnetic field is given by Equation (10).

(10) $$\nabla \times (\frac{1}{\mu }\nabla \times A)=J$$

Here, we assumed the direction of current is parallel with *z*-axis, we can obtain the magnetic vector potential given by Equation (11)

(11) $$\begin{array}{l} A=\left(\begin{array}{ccc} 0 & 0 & A \end{array}\right)\\ J=\left(\begin{array}{ccc} 0 & 0 & J \end{array}\right) \end{array}$$

where, **A** is the magnetic vector potential, **J** is the current density.

**Figure 10:**
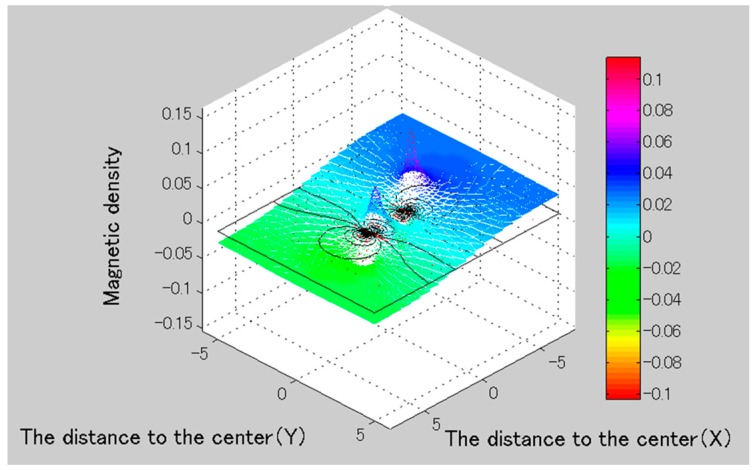
Simulation results of the magnetic flux density.

We can obtain the magnetic field distribution of O-ring magnet, as shown in Figure 10. The simulation result indicated that the O-ring magnet including multi-magnetic potentials is higher magnetic field area than one magnetic potential type [15]. Therefore, the O-ring magnet as the actuator is used to obtain optimal performance in our research.

### 3.2. Principle of the Screw Jet Motion Mechanism

While the magnetically actuated microrobot is moving inside of the rotational magnetic field, the rotational magnetic field generated a magnetic torque and magnetic force acting on the microrobot. Meanwhile, the microrobot rotates synchronously with the changing frequency of rotational magnetic field, due to a pure magnetic moment is generated as a dipole of magnet attempts to align with the local magnetic field. The magnetic force and magnetic torque is defined by Equations (12) and (13) [32].

(12) T=VM×B

(13) F=V(M⋅∇)B

where, *V* and *M* represent the volume and average magnetization of the magnetically microrobot, respectively. ∇ represents a gradient operator.

**Figure 11:**
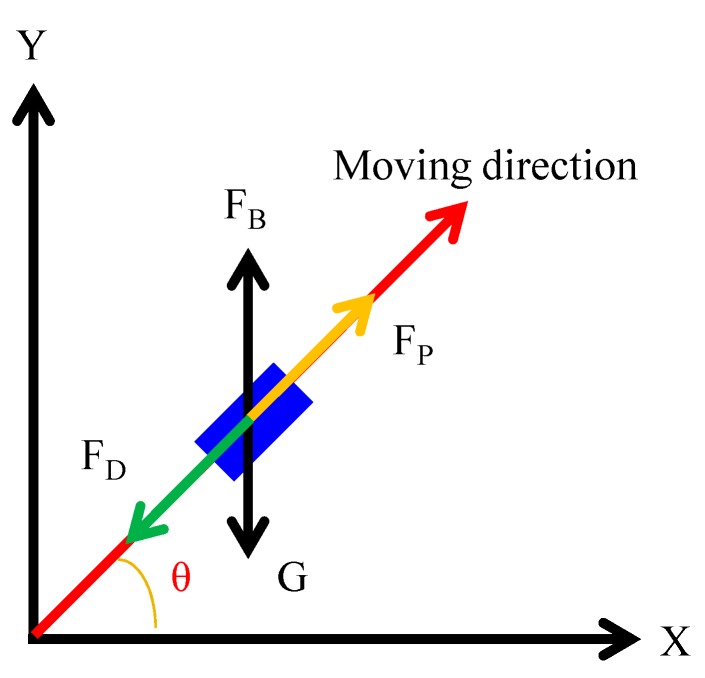
Dynamic model of the microrobot.

**Figure 12:**
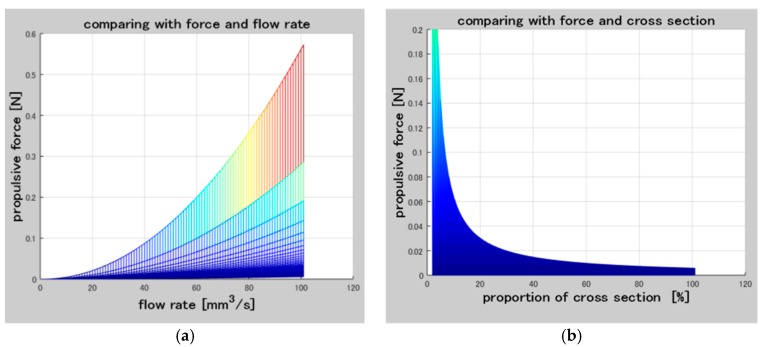
(**a**) Relationship between the propulsive force and flow rate; (**b**) Relationship between the propulsive force and proportion of cross section.

According to the Newton second law, the behavior of microrobots moves in the fluid was analyzed, the equation of the motion of the microrobot in indicated in Equations (13) and (14) [29]. The distribution of force on the magnetically actuated microrobot is simplified including propulsive force, hydraulic resistance, buoyancy and gravity force, as shown in Figure 11. And the relationship between the propulsive force and flow and cross-section of the screw jet mechanism is shown in Figure 12.

(14) $$F_{P}-F_{D}\pm F_{B}\sin\theta \mp G\sin\theta +m\frac{dv}{dt}=0$$

Propulsive force is given by Equation (15).

(15) $$F_{P}=\rho \cdot A\cdot v^{2}-\frac{1}{2}\cdot C_{D}\cdot \rho \cdot A\cdot v^{2}$$

The hydraulic resistance is calculated by Equation (16).

(16) $$F_{D}=C_{D}A\frac{\rho v^{2}}{2}$$

Flow of the screw jet mechanism (Q) is given by Equation (17) [8].

(17) $$Q=a\cdot b\cdot \sqrt{p^{2}+{\left(2\pi r\right)}^{2}}\cdot \Omega$$

where, *F_B_* is buoyancy, resistance, *G* is gravity force, *m* is the mass of microrobot, *v* is moving speed of the microrobot. *F_P_* is the propulsive force, *F_D_* is hydraulic resistance.

## 4. Experimental Results

**Figure 13:**
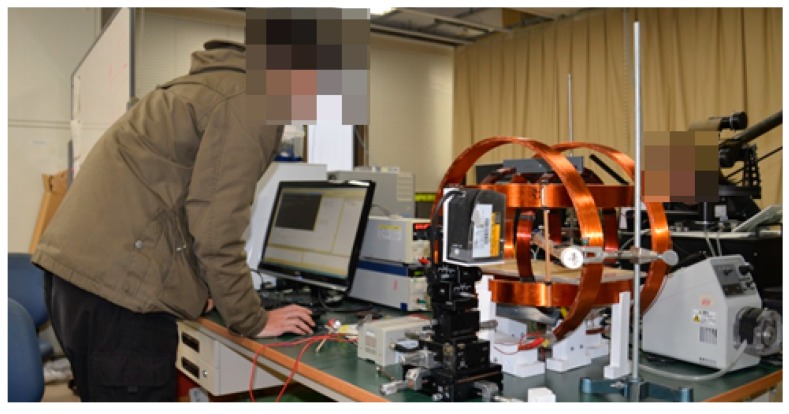
Experimental setup.

An Electromagnetic Actuation (EMA) System was used to evaluate the performance of the magnetically actuated microrobot with screw jet motion, as shown in Figure 13. The magnetically microrobot is placed in a region of interest of the rotational magnetic field generated by the three axes Helmholtz coils. While the signal of the rotational magnetic field is input to the driving system which by an interface developed with Visual Studio 2010, a rotational magnetic field generates. The interface includes control unit, monitor, and an oscilloscope. We can adjust the magnetic changing frequency from 0 Hz to 30 Hz and adjust the direction of the rotational magnetic field to control the position of the microrobot in pipe. Meanwhile, the operator can view the image by a monitor.

**Figure 14:**
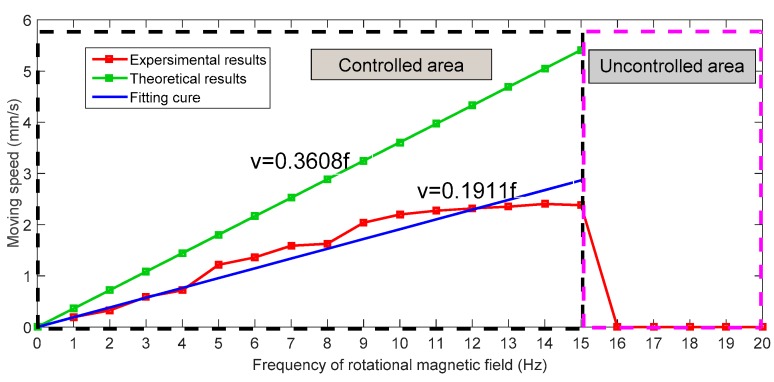
Relationship between the magnetic changing frequency and moving speed.

**Figure 15:**
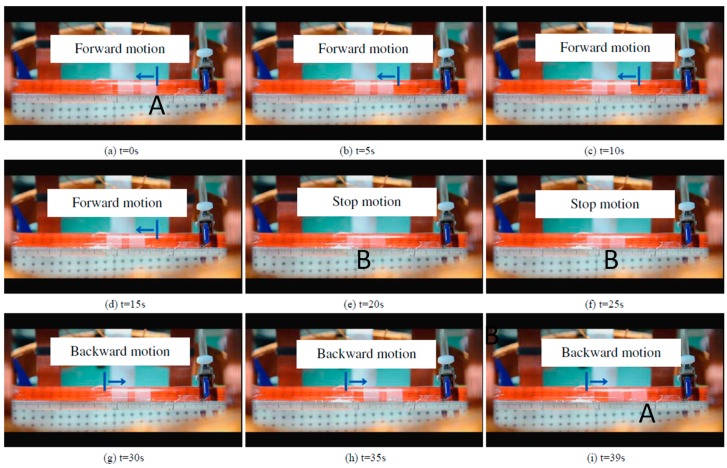
Forward-Stop-Backward motion. (**a**–**d**) shows the forward motion of the microrobot. (**e**,**f**) shows the microrobot stop at a point. (**g**–**i**) shows the backward motion of the microrobot.

**Figure 16:**
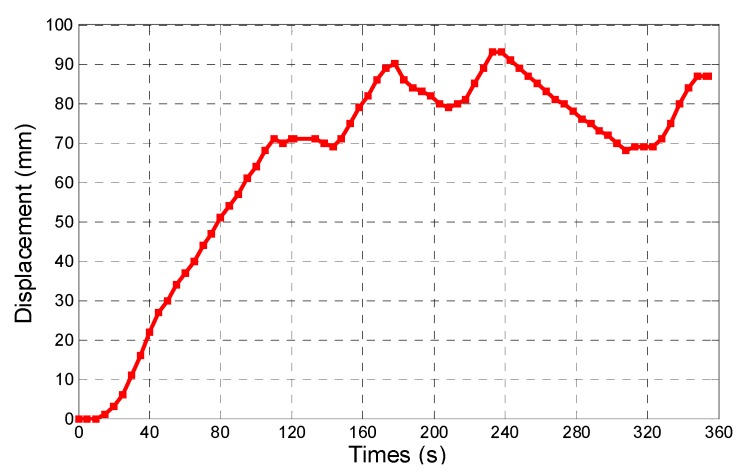
Multiple reciprocating motion.

**Figure 17:**
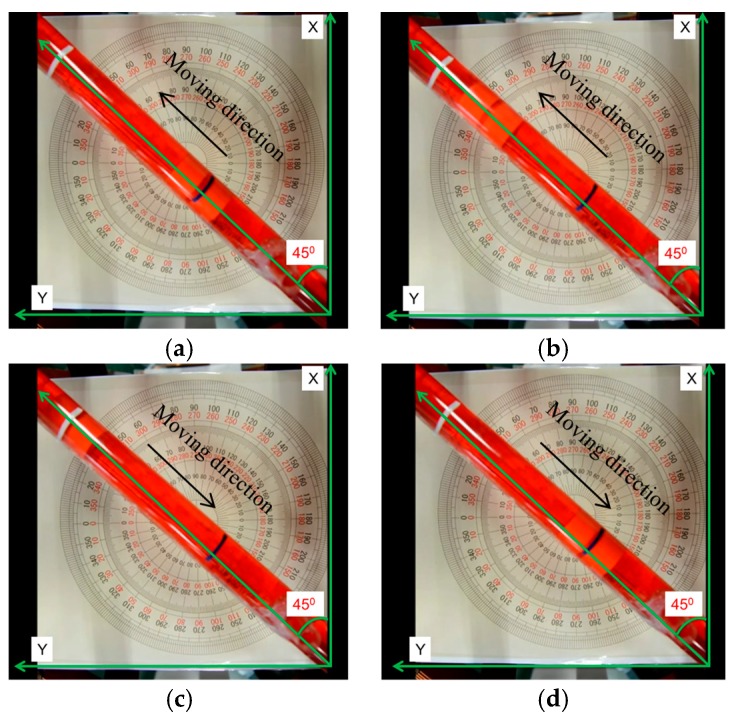
Locomotion direction is 45° direction with *x*-axis (**a**) Phase 1; (**b**) Phase 2; (**c**) Phase 3; (**d**) Phase.

During the experiments, we adjusted the magnetic changing frequency with one frequency each step. The maximum moving speed of the microrobot is 2.4 mm/s. The experimental results show a linear relationship between magnetic changing frequency and moving speed before 15 Hz, as shown in Figure 14. Because, when the magnetic changing frequency over 15 Hz, the magnetically actuated microrobot cannot rotate continuously, synchronized with the rotating magnetic fields, and it cannot generate enough propulsion to overcome the resistance of fluids [29]. The phenomenon is explained by our previous research. In other word, to obtain the stable movement of the microrobot, we just control the magnetic actuated microrobot move in a controlled area in the medical application. Using our proposed magnetic actuated capsule microrobotic system, the magnetically actuated microrobot with screw jet motion realized the reciprocating motion in the horizontal plane locomotion. Based on the movement principle in the Section 2, we controlled the rotational magnetic field by clockwise rotation in X-Z plane, the microrobot moves forwardly from point A to point B with 20 s, and then the microrobot stop at the point B with 5 s. And then the microrobot continues move. At last, adjusting the rotational magnetic field by counter-clockwise rotation, the microrobot moves to point A at 39 s. The experimental results are shown in Figure 15. And then, we designed a multiple reciprocating motion to simulate the process of clinical diagnosis. For example, the microrobot should stop or backward at an area to diagnose. The experimental results are shown in Figure 16. Firstly, the microrobot stop at a point and the microrobot move forward with a uniform motion in the diagnose area (70–90 mm). When the microrobot reach to the diagnose area, the microrobot makes a reciprocating motion to obtain the results (*T* = 120–320 s), and then microrobot move to another diagnose. The magnetically actuated microrobot also realized the movement in any direction in the horizontal plane by our proposed control method in Section 2, which generates any rotational magnetic field of perpendicular to moving direction. The Figure 17 shows one of movements that the locomotion direction is 45° direction with *x*-axis in horizontal plane.

**Figure 18:**
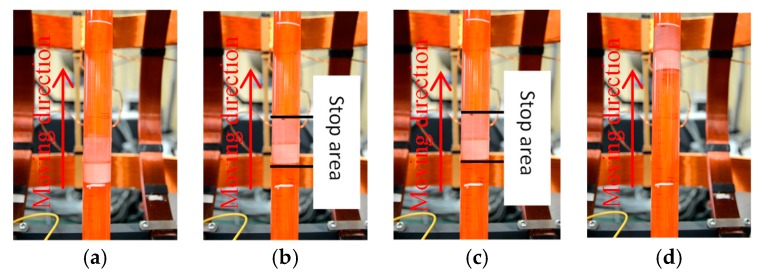
Experimental setup (**a**) Phase 1; (**b**) Phase 1; (**c**) Phase 1; (**d**) Phase 1.

In addition, we carried out the movement of the microrobot on the vertical plane, as shown in Figure 18. Firstly, the microrobot free falls under the action of gravity and resistance, it means that the microrobot moves without the rotational magnetic field. And then, we adjusted the rotational magnetic field frequency to obtain the propulsive force. There are three kinds of phenomenon during the experiments. When the propulsive force is larger than the gravity and resistance, the microrobot can move upward. When the propulsive force is equal the gravity and resistance, the microrobot can stop at a point. And when the When the propulsive force is less than the gravity and resistance, the microrobot can move downward.

## 5. Conclusions

In this paper, we focused on the performance evaluation of a magnetically actuated capsule microrobotic system which consists of only three stationary pairs of Helmholtz coils. We compared two kinds shape of the Helmholtz coils, circular Helmholtz coils, and square Helmholtz coils. The Helmholtz coils produced magnetic flux density of the circular Helmholtz is 1.11 times larger than the square Helmholtz coil under conditions of the same radius. And we proposed a magnetically actuated microrobot with screw jet motion. We analyzed the magnetic field of the O-ring magnet by the Finite Element Method to obtain optimal performance of the microrobot. We confirmed that the magnetically actuated microrobot realized the basic motion using our proposed microrobotic system, such as forward motion and backward motion in horizontal plane and vertical plane. Meanwhile, by changing the frequency of rotational magnetic field, the moving speed of the microrobot was adjusted. In the future, we will focus on how to scale down the size of the microrobot and design some experiments to realize the movement in a confined tube. In addition, we develop a positing system for the microrobot, such as, using a vision module to detect the positing for the microrobot.

## Figures and Tables

**Table 1:** Specifications of the microrobot.

Microrobot	Parameter
Length of microrobot (mm)	35
Radial of microrobot (mm)	16
Weight of microrobot (g)	4.248
Magnetization on direction	Radial
Radial of magnet (mm)	5
Weight of magnet (g)	1.036
Material of body	Polythene Plastic
Material of screw structure	Polythene Plastic
